# Statistical and machine learning based platform-independent key genes identification for hepatocellular carcinoma

**DOI:** 10.1371/journal.pone.0318215

**Published:** 2025-02-05

**Authors:** Md. Al Mehedi Hasan, Md. Maniruzzaman, Jie Huang, Jungpil Shin

**Affiliations:** 1 Department of Computer Science & Engineering, Rajshahi University of Engineering & Technology, Rajshahi, Bangladesh; 2 Statistics Discipline, Khulna University, Khulna, Bangladesh; 3 School of Computer Science and Engineering, The University of Aizu, Aizuwakamatsu, Fukushima, Japan; Instituto do Cancer do Estado de Sao Paulo / University of Sao Paulo, BRAZIL

## Abstract

Hepatocellular carcinoma (HCC) is the most prevalent and deadly form of liver cancer, and its mortality rate is gradually increasing worldwide. Existing studies used genetic datasets, taken from various platforms, but focused only on common differentially expressed genes (DEGs) across platforms. Consequently, these studies may missed some important genes in the investigation of HCC. To solve these problems, we have taken datasets from multiple platforms and designed a statistical and machine learning-based system to determine platform-independent key genes (KGs) for HCC patients. DEGs were determined from each dataset using limma. Individual combined DEGs (icDEGs) were identified from each platform and then determined grand combined DEGs (gcDEGs) from icDEGs of all platforms. Differentially expressed discriminative genes (DEDGs) was determined based on the classification accuracy using Support vector machine. We constructed PPI network on DEDGs and identified hub genes using MCC. This study determined the optimal modules using the MCODE scores of the PPI network and selected their gene combinations. We combined all genes, obtained from previous studies to form metadata, known as meta-hub genes. Finally, six KGs (CDC20, TOP2A, CENPF, DLGAP5, UBE2C, and RACGAP1) were selected by intersecting the overlapping hub genes, meta-hub genes, and hub module genes. The discriminative power of six KGs and their prognostic potentiality were evaluated using AUC and survival analysis.

## Introduction

Hepatocellular carcinoma (HCC) is the leading cause of liver cancer-related deaths, with its mortality rate steadily increasing throughout the world. [1]. More than 80% of liver cancers are attributed account for HCC [2], with its higher percentages were found in men than women [3]. Typically diagnosed between 30 and 50 years [3]. HCC is strongly linked to various factors, including hepatitis (B/C) virus infections, obesity, abuse of alcohol consumption, smoking, and type 2 diabetes [4]. Typically diagnosed Hepatitis B, in particular, is noted as a significant risk factor [5]. Despite therapeutic advancements including radiation, chemotherapy, and targeted therapy, patients with HCC still face low survival rates [6]. Late diagnosis of HCC significantly contributes to rising incidence and mortality rates, underscoring the critical need for early detection methods and improved access to treatment to mitigate these risks [7, 8].

Today, statistical model-based bioinformatic analysis is effective in identifying key genes (KG) and their correlated molecular pathways in various cancers, including HCC [6, 9–59]. It helps in understanding the molecular mechanisms underlying cancer development and progression, which play a vital role in identifying targets for targeted therapy and improving patient outcomes. For instantaneous, Zhao et al. [12] determined seven hub genes and also their molecular pathway (gene ontology (GO) terms and Kyoto Encyclopedia of Genes and Genomes (KEGG) pathway). Liu et al. [13] also introduced ten prognostic biomarkers that played an important role of developing HCC. Li et al. [16] proposed five genes, also strongly correlated with patients with HCC.

In existing studies, researchers have attempted to propose promising biomarkers for detecting HCC. The majority of the existing studies conducted their research on genetic datasets that were taken from a particular platform [9, 12, 13, 19, 41, 59] or different platforms but they considered only common differentially expressed genes (DEGs) among those platforms [14, 18, 23, 24]. As a result, both types of studies may be missed important genes in their analysis. So, there is a scope to identify platform-independent KGs by including all important genes from all microarray platforms. These existing Most existing studies derived hub genes or key genes from PPI network. Recently, machine learning (ML)-based approaches have widely used and gained more popularity for identifying potential biomarkers from genetic data [60, 61]. So, the integration of machine learning, statistical models, and bioinformatics can indeed the evidence and confidence in identifying a biomarker to be a KG.

**Fig 1:**
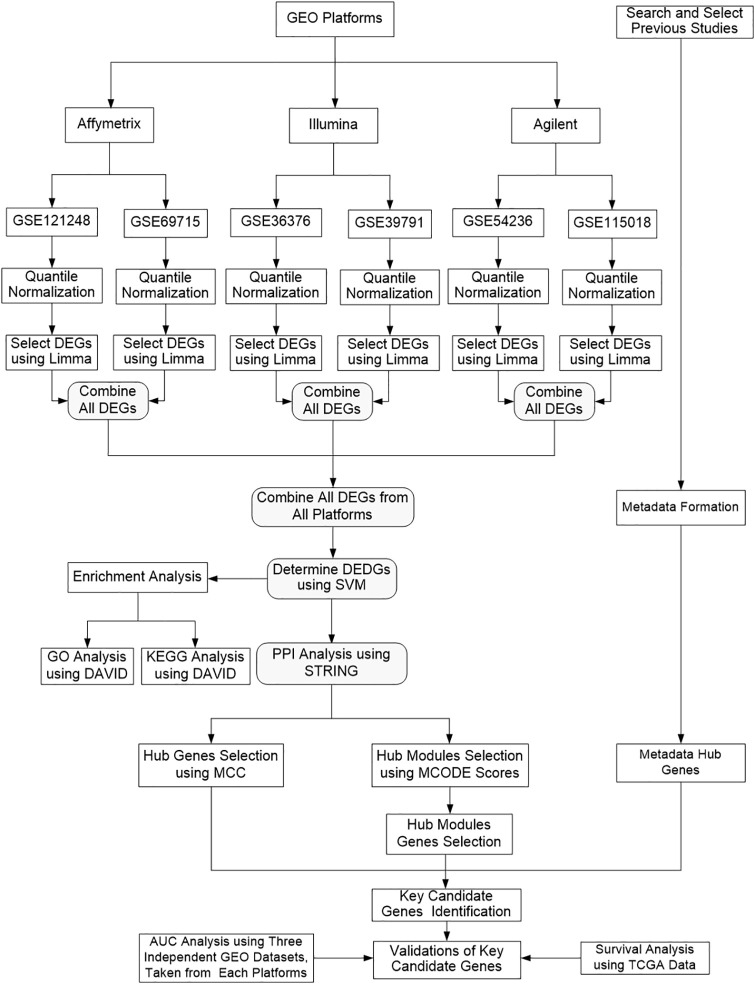
Overall flowchart of the proposed methodology in identifying KGs.

In this study, six different datasets from three microarray platforms were used as training datasets to identify KGs using statistical and ML-based approaches. To determine KGs, we performed several experiments. Firstly, DEGs were identified individually from each dataset. Secondly, individual combined DEGs (icDEGs) were identified from two datasets of each platform. Thirdly, grand combined DEGs (gcDEGs) were combined and identified across all platforms. Fourth, differentially expressed discriminative genes (DEDGs) were determined from gcDEGs by support vector machine (SVM). Fifthly, the Cluster profile was performed for the enrichment analysis. The PPI network was constructed using STRING and visualized through Cytoscape as well as determined hub genes using maximal clique centrality (MCC). Module analysis was performed using Molecular Complex Detection (MCODE) and determined the significant modules and their associated significant genes. To incorporate the inference of other existing studies, metadata were formed by combing all genes from previous studies and identified meta-hub genes. Finally, the KGs were identified by intersecting common genes from hub genes, significant module genes, and meta-hub genes. These identified KGs demonstrate greater discriminatory power than other genes due to their statistical significance, robust validation, association with critical pathways, enhanced performance in predictive models, and unique identification through necessity analysis. Another three independent test datasets, taken from three microarray platforms were used to validate these KGs and confirmed that these identified KGs have more discriminative power. So, the flowchart of our proposed methodology for determining KGs in HCC patients is more clearly explained in Fig 1.

## Materials and methods

### Microarray data acquisitions

Ten publicly available datasets were used to conduct the study. Nine datasets with GEO accession: GSE121248 [62], GSE69715 [63], GSE14520 [64], GSE36376 [65], GSE39791 [66], GSE87630 [67], GSE54236 [68], GSE115018 [69], and GSE47197 was extracted from the GEO database (www.ncbi.nlm.nih.gov/geo/). Another TCGA-LIHC dataset was extracted from the TCGA database (https://portal.gdc.cancer.gov/). Three GEO datasets (GSE121248, GSE69715, and GSE14520) were derived from the Affymetrix platform (GPL570 and GPL571). The GSE36376, GSE87630, and GSE39791 datasets were from Illumina platform (GPL10558 and GPL6947) and another three datasets (GSE54236, GSE115018, and GSE47197) were from Agilent platform (GPL6480, GPL20115, and GPL16699). The GSE115018 dataset with GPL20115 Agilent platform had two types of marker-IncRNA + mRNA microarray. This study analyzed only the mRNA data of GSE115018. The Probe ID mapped to gene symbols from each platform and incorporated them into the dataset matrix for each dataset. Six datasets (GSE121248, GSE69715, GSE36376, GSE39791, GSE54236, and GSE115018) were employed to identify the KGs. Another four independent datasets (GSE14520, GSE87630, GSE47197, and TCGA-LIHC) were used to validate the KGs. The list of utilizing ten datasets was explained more clearly in Table 1.

**Table 1:** List of utilized HCC-based microarray gene expression datasets.

Datasets	Platform Number	Platform Name	Samples	HCC	CC
GSE121248 [62]	GPL570	Affy.	107	70	37
GSE69715 [63]	GPL570		103	66	37
GSE14520 [64]	GPL571		445	225	220
GSE36376 [65]	GPL10558	Illum.	433	240	193
GSE39791 [66]	GPL10558		144	72	72
GSE87630 [67]	GPL6947		94	64	30
GSE54236 [68]	GPL6480	Agil.	161	81	80
GSE115018 [69]	GPL20115		24	12	12
GSE47197	GPL16699		124	61	63
TCGA-LIHC	–	–	424	374	50

Affy.: Affymetrix; Illum: Illumina; and Agil.: Agilent; CC: Control.

### Effects of platform independent vs platform dependent data analysis

**Fig 2:**
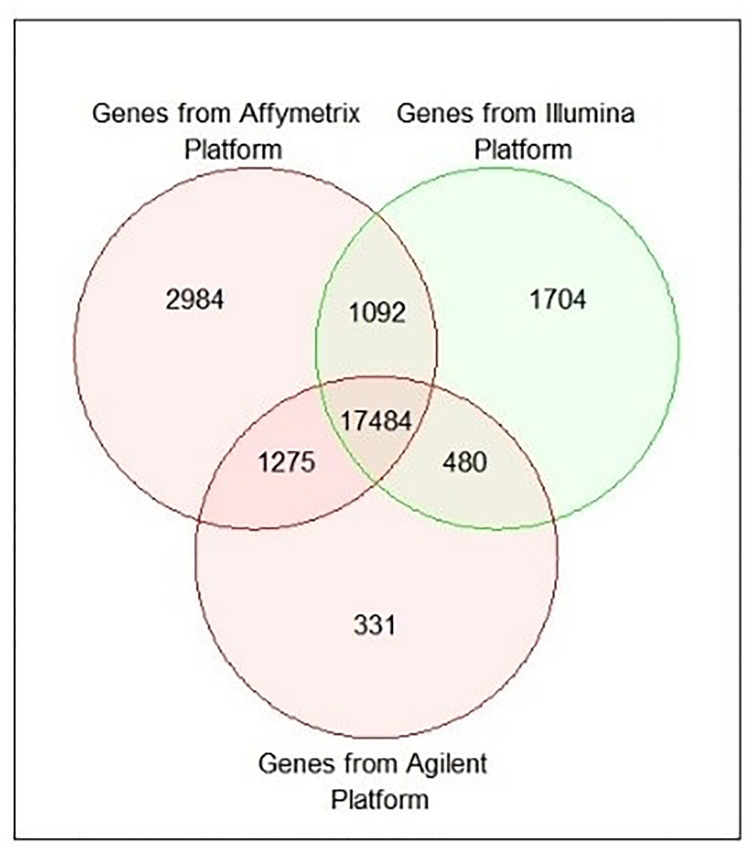
Identification of shared gene among genes from Affymetrix, Illumina, and Agilent platforms.

Usually, microarray gene expression datasets are provided in a rich public repository, like GEO [70] to study various types of disease, including cancer. Generally, microarray datasets have been formed using different platforms, namely Affymetrix, Illumina, and Agilent. The number of genes is not equal of these platforms. For example, there are 54675 probe ids presented in the Affymetrix platform, 47323 probe ids in the Illumina platforms, and 41000 probe ids in the Agilent platform. There were 22835, 20760, and 19570 unique gene symbols found in Affymetrix, Illumina, and Agilent platforms, respectively as shown in Table 2 and their Venn diagram is presented in Fig 2. The majority of the existing studies conducted their research on genetic datasets that were taken from a particular platform [9, 12, 13, 19, 41, 59] or different platforms but they considered only common DEGs among those platforms [14, 18, 23, 24]. As a result, both types of studies missed some important genes in their analysis. For instance, 17484 common genes were determined from Affymetrix, Illumina, and Agilent platforms. If we considered only these 17484 common genes for the analysis, the remaining important genes may be eliminated from consideration to be KGs. On the other hand, if we considered only genes (2984+1092+17484+1275 = 22835) (see in Fig 2) from the Affymetrix platform, rest of the important genes may be also missed from the analysis. To solve these problems, we have taken datasets from three different microarray platforms and designed a statistical and ML-based computational approach for identifying platform-independent KGs for HCC by considering all important genes.

**Table 2:** List of gene symbols in Affymetrix, Illumina, and Agilent platforms.

	Platform Name		
	Affy.	Illum.	Agil.
Probe ID	54675	47323	41000
Probe ID Present & Gene Symbol Blank	9557	16057	10277
Unique Gene Symbol	22835	20760	19570
Common Gene Symbols	17484		

### Identification of DEGs from individual GEO dataset

This study performed log_2_ transformation as well as quintile normalization of the selected GEO datasets. DEGs were obtained using the “limma” based R package (version 4.1.2) [71]. Moreover, an empirical Bayes procedure was implemented for analyzing the gene expression difference between HCC and normal tissues [72]. At the same time, the |log_2_FC| and adj. *p*-values were computed for correspondence each gene. This study converted probe ids into gene symbols using the “Bioconductor annotation” package [73] and found a unique gene symbol matched with multiple probe IDs. We selected the gene symbol with its correspondence expression value, which gave the minimum adjusted p-value. The genes which have |log_2_FC|≥1.2 and adj. *p*-value < 0.01 were chosen as DEGs [74, 75]. Moreover, we used ggplot2 package (version 3.3.6) [76] for generating the volcano plot and NMF package (version 0.24.0) [77] for creating the heatmap of DEGs in R.

### Identification of icDEGs from each platform

Generally, GEO microarray datasets were taken from the Affymetrix, Illumina, and Agilent platforms. Most of the existing studies identified common DEGs after identifying DEGs from individual datasets with particular [9, 12, 13, 19, 41, 59] or different microarray platforms and took common DEGs for analysis [14, 18, 23, 24]. As a result, some potential DEGs were missed from the analysis due to platform-independent [78]. To solve these problems, we considered all DEGs, obtained from GEO datasets on the same platforms using the following formula:

$$\begin{array}{c} \text{icDEGs}\;\text{of}\;{\text{Platform}}_{j}=\overset{r_{s}}{\underset{i=1}{⋃}}\text{Identified}\;\text{DEGs}\;\text{from}\;{\text{Dataset}}_{i}\text{of}\;{\text{Platform}}_{j} \end{array}$$ (1)

where, *r*_*s*_ is the no. of GEO datasets (*r*_*s*_ = 2) and *j* is the number of platforms (j = 3).

### Identification of gcDEGs from all platforms

Most of the existing studies were conducted their studies by taking GEO datasets with different platforms. These existing studies determined common DEGs after identifying DEGs, obtained from the GEO dataset with different platforms, and used common DEGs for analysis. As a result, more significant DEGs were also missed or ignored from the analysis [78]. In this case, we identified grand combined DEGs (gcDEGs), obtained from three microarray platforms (Affymetrix, Illumina, and Agilent) using the following formula:

$$\begin{array}{c} \text{gcDEGs}\;=\overset{r_{d}}{\underset{i=1}{⋃}}\text{Combined}\;\text{DEGs}\;\text{from}\;{\text{Platforms}}_{i} \end{array}$$ (2)

Where, *r*_*d*_ is the number of platforms (here, *r*_*d*_ = 3). Here, “Combined DEGs” refers to DEGs identified by integrating data from multiple datasets across platforms through bioinformatics analyses.

### SVM based DEDGs identification

SVM is widely used for classification, regression, gene or feature selection, and outlier detection methods. SVM aims to find a hyperplane in a high-dimensional space [79] that effectively separates different classes (HCC versus control) by maximizing the following formulae:

$$\begin{array}{c} f\left(z\right)=\;\sum_{i=1}^{n}\alpha_{i}K\left(z_{i},\;z_{j}\right)+b \end{array}$$ (3)

This study used radial basis function (RBF) for SVM and tuned their correspondence hyperparameters (cost (C) and gamma (*γ*)) using a grid search approach. In our study, SVM model was employed to determine DEDGs and its computational formula is explained more clearly as follows:

**Step 1:** Select 80% of the dataset as a training set and remaining datasets for test sets.
**Step 2:** Select one gene from a list of identifying gcDEGs from all platforms.
**Step 3:** Trained SVM with RBF kernel with 5-fold CV.
  (a) Calculate accuracy for a DEG if it is found in one dataset.
  (b) Calculate the average accuracy for a DEG If it is found in multiple datasets.
**Step 4:** Repeat **Step 1** to **Step 3** for all gcDEGs (2,264) obtained from all platforms.
**Step 5:** Sort the DEGs according to their accuracy or average accuracy in descending order of magnitude.
**Step 6:** Take DEGs as DEDGs whose provided the accuracy or average is of more than 95.0%.

### Enrichment analysis

Enrichment analysis was performed on DEDGs by the cluster profile based package (version 3.10.0) [80] and “org.Hs.e.g.db” annotation based package (version 3.10.0) [81] in R to understand the molecular the progression and mechanisms of patients with HCC. The cluster profile was used to determine the GO-based terms including biological process (BP), cellular component (CC), and molecular function (MF), and also their associated pathways which were involved in HCC. The KEGG database was chosen to match the datasets for biological interpretations. The optimal GO and KEGG pathways was selected using p-value (<0.05).

### Analysis of PPI network and hub gene identification

In our study, we constructed the protein-protein interaction (PPI) networks, data from STRING-based biological database (version 11.5) (www.string-db.org) which was typically used to gather known protein interactions [82]. The proteins are represented as nodes, and the interactions between them as edges. The PPI network can be constructed based on experimental data or computational predictions, which results in a graph where the nodes (proteins) are linked by edges that signify physical or functional interactions. In previous studies, thresholds like 0.30, 0.50, and 0.70 have been applied when analyzing PPI networks. Interactions with lower confidence scores are often unreliable and may represent false positives. By setting the threshold to 0.70, we can filter out these false positives, ensuring that the remaining interactions are more likely to be biologically meaningful. Therefore, for constructing the PPI network, we have used a confidence score threshold of 0.70 and set the maximum number of interactors to ‘0’ to further refine the network. Cytoscape (version 3.9.1) [83] was employed to generate PPI network within DEDGS. Additionally, Cytoscape-plugin cytoHubba was employed to rank the DEDGs (nodes) in the PPI network using the eleven topological measures such as degree, edge percolated component, MCC, and six centralities (Bottleneck, EcCentricity, Closeness, Radiality, Betweenness, and Stress) based on shortest paths [84]. Among the eleven methods, MCC has a better performance on the precision of predicting essential proteins from PPI network [84]. In our study, We chose MCC as it has been shown to outperform other measures in predicting essential proteins based on the existing studies such as Chin et al. [84], where MCC demonstrated superior precision in identifying critical nodes in biological networks. Consequently, we ranked the MCC values from the largest to lowest and selected the top 20 DEDGs as hub genes, which was used to determine KGs for patients with HCC.

### Hub modules and identifying their associated genes

MCODE (Molecular Complex Detection) method is used for clustering PPI networks into highly interconnected subgraphs. MCODE was chosen due to its ability to detect functional protein complexes efficiently. It is easy to implement, works well for large networks, and is sensitive to local density variations, making it highly suitable for detecting biologically relevant sub-networks in PPI data. We used degree (2=), k-score (=2), nodes score (=0.2), and max depth (=100) based criteria for performing module analysis [85, 86]. The optimal modules (om) were chosen with MCODE scores (≥6) and the no. of nodes (≥6) and selected hub module genes using the following formula:

$$\begin{array}{c} \text{Hub}\;\text{Module}\;\text{Genes}=\overset{\text{om}}{\underset{i=1}{⋃}}{\text{Genes}\;\text{from}\;\text{Module}}_{i} \end{array}$$ (4)

### Meta hub genes formation from existing studies

Some previous studies on the identification of DEGs for HCC were summarized from three different platforms (Affymetrix, Illumina, and Agilent) [6, 9–59] and listed their hub genes [6, 9–59], known as meta-hub genes. These meta-hub genes were calculated using the following formula:

$$\begin{array}{c} \text{Meta}\;\text{Hub}\;\text{Genes}\;=\overset{MHG}{\underset{i=1}{⋃}}{\text{Hub}\;\text{Genes}\;\text{from}\;\text{Existing}\;\text{Study}}_{i} \end{array}$$ (5)

where, “MHG” is the no. of existing studies from identifying hub genes (here, M = 48). These meta-hub genes were also used to identify KGs.

### Identification of KGs

The following formula was used to determine KGs for HCC:

$$\begin{array}{c} \text{KGs}=\overset{K}{\underset{i=1}{⋃}}{\text{Genes}\;\text{obtained}\;\text{from}\;\text{Identification}\;\text{based}\;\text{Methods}}_{i} \end{array}$$ (6)

where, K is the no. of optimal gene determination approaches (Here, K = 3). This study considered three identification-based approaches (hub genes, significant module genes, and meta-hub genes) to determine the important genes for HCC.

### Validation of KGs

#### Discriminative capability analysis using AUC

Four independent datasets (GSE14520, GSE87630, GSE47197, and TCGA-LIHC) were utilized to validate the KGs for HCC. Among them, three independent datasets were taken from three platforms (Affymetrix, Illumina, and Agilent platforms). The detailed descriptions of these data sets are presented in Table 1. Logistic regression model was used and their respective AUC values [87] to determine the discriminative power of the KGs.

#### Survival analysis

We extracted 374 HCC patients from the TCGA database for survival analysis. To perform survival analysis, patients with HCC were divided into low-risks and high-risks groups using median of their respective gene expression value. The relationship between the KGs and survival status (alive/dead) was assessed using cox proportional hazard model. We performed the survival analysis of KGs using the “Survfit” based R package [88] (<0.05).

### Necessity analysis of platform-independent KG identification

Like existing studies, this study determined the common DEGs from the GO based datasets of the same platform and common DEGs from datasets of all platforms in order to show whether it is possible to identify six KGs by following the procedure of existing studies. In this work, we performed the independent analysis in order to present or absence of KGs into five different viewpoints: (i) KGs vs. identified common DEGs from two datasets of Affymetrix platform, (ii) KGs vs. identified common DEGs from two datasets of Illumina platform, (iii) KGs vs. identified common DEGs from two datasets of Agilent platform, (iv) KGs vs. identified common DEGs from six datasets of all platforms, and (v) KGs vs. identified grand combined DEGs from six datasets of all platforms.

## Experimental results

### Identification of DEGs from individual dataset

**Fig 3:**
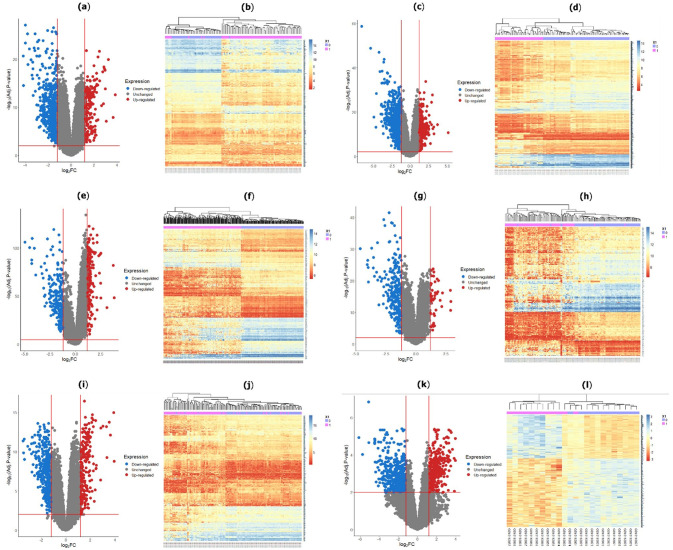
Volcano plot and heatmap of DEGs for each dataset: (a)–(b) for GSE121248; (c)–(d) for GSE69715; (e)–(f) for GSE36376; (g)–(h) for GSE39791; (i)–(j) for GSE54236; (k)–(l) for GSE115018 datasets. Dodger blue, Gray, and fire brick color represent downregulated, no significant, and upregulated DEGs, respectively.

The “limma” based R-package was utilized to obtain individual DEGs from six GEO datasets (GSE121248, GSE69715, GSE36376, GSE39791, GSE54236, and GSE115018). Using filtering criteria |log_2_FC|≥1.2 and adj. p-value<0.01, a total of 645 DEGs, 1084 DEGs, 377 DEGs, 279 DEGs, 563 DEGs, and 734 DEGs were determined from GSE121248, GSE69715, GSE36376, GSE39791, GSE54236, and GSE115018 datasets, respectively. The volcano plots and their correspondence heatmaps of identifying DEGs for individual datasets were depicted in Fig 3.

### Identification of icDEGs from each platform

**Fig 4:**
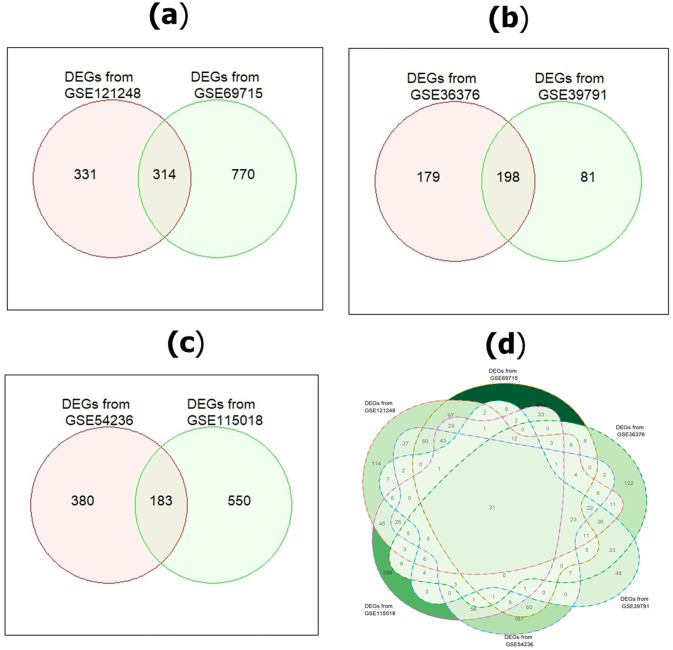
Identification of icDEGs from two datasets of each platform: (a) DEGs of GSE121248 and GSE69715 obtained from Affymetrix; (b) DEGs of GSE36376 and GSE39791 obtained from Illumina; (c) DEGs of GSE54236 and GSE115018 obtained from Agilent platforms; (d) Common DEGs identification from six datasets (GSE121248, GSE69715, GSE36376, GSE39791, GSE54236, and GSE115018) of all platforms.

We identified icDEGs from two datasets of each platform. The DEGs were identified from these three platforms, and their Venn diagrams are illustrated in Fig 4. As shown in Fig 4a, a total of 1415 (331+314+770) icDEGs were obtained from Affymetrix, 458 (179+198+81) icDEGs were from Illumina, and 1,113 (380+183+550) icDEGs were from Agilent platform, respectively. Moreover, we also performed the intersection among DEGs, obtained from six datasets across three microarray platforms (see in Fig 4d). As shown in Fig 4d, we observed that only 31 DEGs were common among six GEO datasets. If we consider these 31 common DEGs, some potential DEGs may be missed from the analysis. For this reason, we considered icDEGs from two datasets of each platform.

### Identification of gcDEGs from all platforms

**Fig 5:**
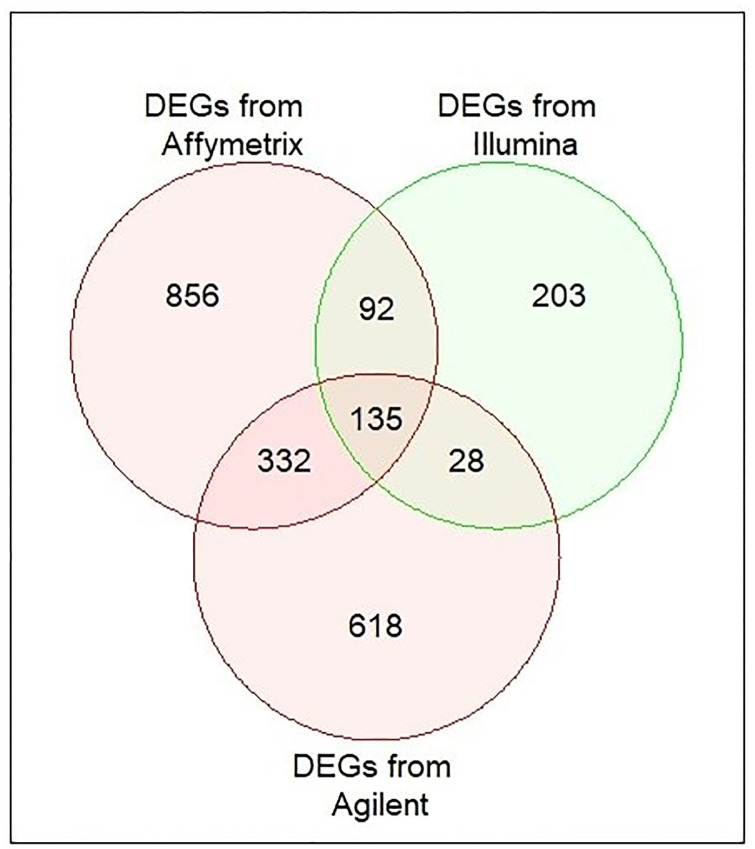
Identification of combined all DEGs (2265) from Affymetrix, Illumina, and Agilent platforms.

We also performed the intersection among DEGs, obtained from Affymetrix, Illumina, and Agilent platforms, and their Venn diagrams are depicted in Fig 5. As shown in Fig 5, we obtained 135 common DEGs from icDEGs of Affymetrix, Illumina, and Agilent platforms. If we also considered these 135 common DEGs among all platforms, some potential DEGs may also be missed from the analysis. In this case, we considered all 2264 (856+92+203+135+332+28+618) DEGs, called as gcDEGs. These 2265 gcDEGs were used for further analysis.

### SVM based DEDGs identification

**Fig 6:**
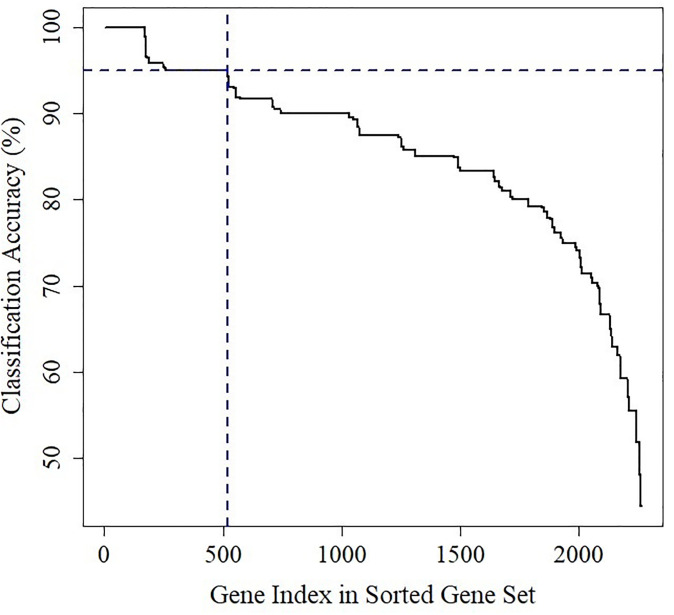
Classification accuracy of all gcDEGs for identifying DEDGs.

SVM was implemented on gcDEGs to determine DEDGs for HCC and computed classification accuracy for each gene of gcDEGs from all platforms. If the DEGs were found in multiple datasets, then compute the average classification accuracy. The identification procedure of DEDGs using SVM is more clearly explained in the methodology section. After that, gcDEGs were sorted in descending (largest to lowest) based on classification accuracy as shown in Fig 6. Therefore, 518 DEDGs were identified because their accuracy or average accuracy was more than 95.0%.

### Enrichment analysis on DEDGs

**Fig 7:**
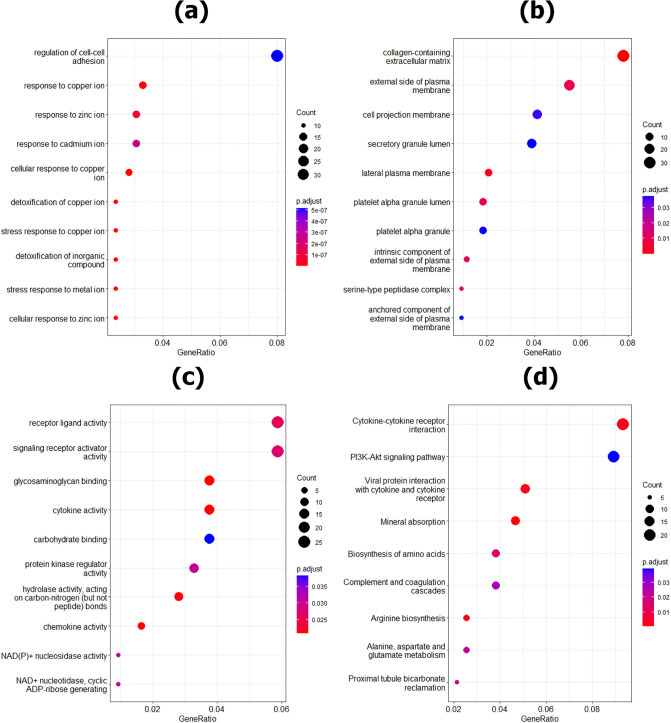
Enrichment analysis for DEDGS: (a) BP; (b) CC, (c) MF, and (d) KEGG pathways.

Enrichment analysis was performed on 518 DEDGs to understand the molecular mechanism of developing HCC. The DEDGs were significantly enriched in a total of 309 BP-based GO terms, 17 CC-based GO terms, 18 MF-based GO terms, and 9 KEGG-based pathways (p<0.05). The top ten BP, CC, and MF-based GO terms and nine KEGG pathways were illustrated in Fig 7.

### PPI network construction and hub gene selection

**Fig 8:**
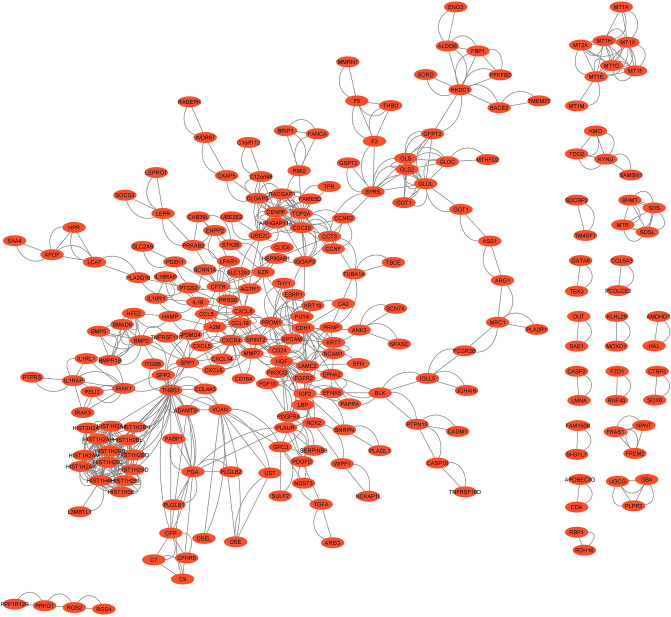
Network analysis of DEDGs with 233 nodes and 748 edges.

A PPI network was constructed on 518 DEDGs using STRING and is displayed in Fig 8. A total of 233 nodes and 748 edges were connected in the PPI network with an average clustering coefficient of 0.283, network density of 0.02, and connected components of 19. The scores of MCC were computed from the PPI network using the Cytoscape plug-in cytoHubba and then, sorted the genes based on MCC. At the same time, the top 20 DEDGs (CDC20, TOP2A, CENPF, DLGAP5, UBE2C, ARHGAP11A, RACGAP1, HIST1H2AJ, HIST1H2AH, HIST1H2AM, HIST1H2AK, HIST1H2BO, HIST1H4H, HIST2H2AB, HIST1H2BJ, HIST1H2BB, HIST1H3E, HIST1H2AD, HIST1H2BI, and HIST1H2BL) were selected, which were treated as hub genes in this work.

### Hub modules and its associated genes identification

**Fig 9:**
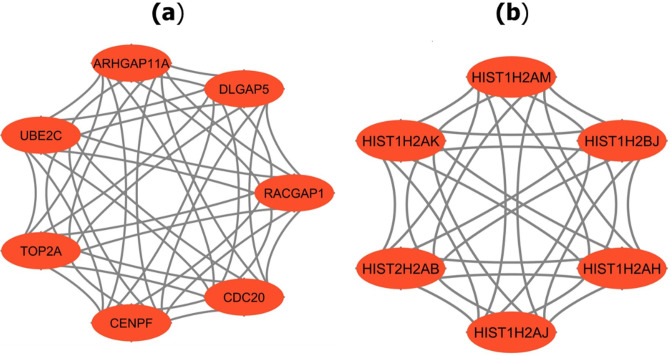
PPI network of two hub modules: (a) Module 1, (b) Module 2.

MCODE analysis was employed to module analysis and identified 18 modules with MCODE scores from 2.8 to 7.0. We observed that module 1 and module 2, provided the MCODE scores and nodes of more than 6. These two modules were considered as significant models and their PPI networks were shown in Fig 9. There were 7 nodes and 42 edges in module 1, whereas module 2 contained 6 nodes and 30 edges. To make hub module, this study combined module 1 and module 2 and identified their respective 13 genes, known as significant module genes.

### Meta hub genes determined from existing studies

Forty eight previous studies were summarized related to gene identification for patients with HCC [6, 9–59]. To make align with other previous studies, we compiled metadata by identifying all hub genes from earlier research and determining meta hub genes. The identified hub genes and corresponding metadata are listed in Table 3. Finally, this study identified 138 hub genes from previous studies, which were used to identify KGs of HCC.

**Table 3:** Meta-hub genes formation from previous studies.

SN	Authors	Platform Name	NHG	Associated Hub Genes	SN	Authors	Platform Name	NHG	Associated Hub Genes
1	Maddah et al. [9]	Affy.	10	BUB1, DLGAP5, CDCA8, ASPM, POLQ, WDHD1, CENPE, HELLS, TRIP13, DEPDC1	25	Chen et al. [39]	Affy.	11	RRM2, ECT2, NDC80, ASPM, CDK1, CCNB1, PRC1, KIF20A, TOP2A, DTL, PBK
2	Zhao et al. [12]	Affy.	7	CCNA2, CDK1, CCNB1, MAD2L1, RRM2, NDC80, TOP2A,	26	Yu et al. [36]	Affy.	6	TOP2A, CDC6, MAD2L1, CHEK1, CCNB1, UBE2C
3	Liu et al. [13]	Affy.	10	CYP3A4, AOX1, UGT1A4, UGT1A6, DK1, UGT2B15, C CCNB2, CCNB1, CDC20, MAD2L1,	27	Kakar et al. [37]	Affy.	10	CDK1, CCNB1, CCNA2, CCNB2, NDC80, BUB1, CDC20, NCAPG, BUB1B, MAD2L1
4	Meng et al. [14]	Affy.\Illum.\Agilent	11	CDK1, CDC20, CCNB2, CCNB1, CCNA2, TOP2A, MELK, KIF20A, PBK, TPX2, AURKA	28	Ji et al. [38]	Affy.\Agil.	10	CDK1, PBK, ASPM, CCNB1, CCNB2, NDC80, AURKA, KIF2C, TPX2, CENPF
5	Rosli et al. [15]	Affy.	21	CDK1, MAD2L1, TOP2A, CCNA2, CCNB1, CCNB2, KIF11, NCAPG, TTK, AURKA, CDC20, RRM2, NDC80, CENPA, MELKPBK, BUB1B, PRC1, DTL, NUSAP1, KIF2C	29	Zhou et al. [35]	Affy.	15	DTL, RACGAP1, CDK1, CCNB1, ECT2, NEK2, BUB1B, ASPM, HMMR, PBK, TOP2A, RRM2, CDKN3, PRC1, ANLN
6	Zhang et al. [6]	Affy.	10	GMPS, ACACA, KRAS, BCL2, ALB, TGFB1, EGFR, STAT3, ERBB2, CD8A	30	Qiang et al. [40]	Affy.	10	CDK1, CDC20, CCNB2, BUB1, CCNB1, NDC80, BUB1B, CENPF, NUF2, MAD2L1
7	Li et al. [16]	Affy.	5	SPP1, IGF1, LGALS3, COL1A2, LPA	31	He et al. [47]	Affy.	4	CDK1, RRM2, PBK, ASPM
8	Li et al. [17]	Affy.	8	BUB1, CCNA2, BUB1B, CCNB1, CDK1, CDC20, MAD2L1, CCNB2	32	Zhang et al. [44]	Affy.	10	CCNB1, TOP2A, AURKA, NEK2, NUF2, CENPF, CDKN3, ASPM, PRC1, RACGAP1
9	Tian et al. [18]	Affy.\Illum.	5	CDC20, RRM2, TOP2A, AOX1 UBE2C,	33	Wang et al. [54]	Affy.	5	CDK1, CCNB2, CCNB1, TOP2A, MAD2L1
10	Wan et al. [19]	Agil.	12	GF1, NDC80, IGF2, CDK1, CDCA8, CCNB1, CENPF, BIRC5, NCAPG, CDCA5, SPC25, CENPU	34	Sha et al. [45]	Affy.	14	TOP2A, DTL, HMMR, CCNB1, PBK, NEK2, RACGAP1, CDK1, PRC1, RRM2, BUB1B, ECT2, ANLN, ASPM
11	Zhu et al. [20]	Affy.	10	CDK1, CCNB1, TOP2A, CDC20, PLK1, CCNB2, BIRC5, FOS, AURKA, AURKB	35	Chen et al. [39]	Affy.\Illum.\Agil.	10	TOP2A, PRC1, CCNB2, RACGAP1, CDKN3, AURKA, NUSAP1, ASPM, CDCA5, NCAPG
12	Wang et al. [21]	Affy.	10	TOP2A, ITGA2, CDK1, PLK1, CCNB2, ESR1, AURKA, CCNA2, BUB1, BUB1B	36	Wang et al. [41]	Illum.	10	CDKN3, UBE2C, TOP2A, CDC20, ASPM, PBK, KIF20A, CCNB2, NCAPG, CYP3A4
13	Zhou et al. [22]	Affy.\Illum.\Agil.	9	ASPM, CCNB2, AURKA, MELK, CDKN3, NUSAP1, NCAPG, TOP2A, PRC1	37	Zhang et al. [48]	Affy.\Agil.	10	NEK2, TOP2A, ANLN, CENPF, CDC20, ASPM, CDK1, ECT2, CCNB1, CCNB2
14	Zhang et al. [23]	Affy.\Agil.	10	CDK1, AURKA, CCNB1, KIF11, CCNA2, TOP2A, BUB1B, HMMR, TPX2, CDC45	38	Zhang et al. [50]	Affy.\Illum.	9	ALDH2, CYP2C8, PPTG1, ADH1B, ADH4, CYP2C8, TOP2A, CDC20, CCNB2
15	Nguyen et al. [34]	Affy.	5	TOP2A, NEK2, RRM2, CCNB1, CDK1,	39	Hu et al. [49]	Illum.	4	JUN, MYC, EGR1, CDKN1A
16	Wu et al. [33]	Affy.	8	CDKN3, CCNB1, CDK1, TOP2A, CCNB2, CCNA2, RRM2 PRC1	40	Li et al. [51]	Affy.	15	TOP2A, CCNB1, CDK1, BUB1, CCNB2, CENPF, TTK, KIF2C, MELK, HMMR, CENPE, PBK, KIF4A, KIF20A, DLGAP5
17	Gui et al. [26]	Agil.	4	MT1X, CAP2, BMI1, TACSTD2	41	Cao et al. [52]	Affy.\Illum.	5	MCM3, KIF11, PBK, CHEK1, S100A9
18	Wang et al. [27]	Affy.	10	NDC80, TOP2A, CDK1, AURKA, HMMR, CCNB1, FOXM1, CENPF, PTTG1, CDKN3	42	Zhang et al. [6]	Affy.	10	GMPS, ALB, ACACA, TGFB1, ERBB2, KRAS, BCL2, STAT3, EGFR, CD8A
19	Zhang et al. [30]	Affy.	10	GMPS, ALB, ACACA, TGFB1, ERBB2, KRAS, BBCL2, STAT3, EGFR, CD8A	43	Kim et al. [43]	Affy.	14	ANLN, BUB1B, ASPM, CCNB1, CDKN3, CDK1, ECT2, NEK2, HMMR, PBK, RACGAP1, PRC1, TOP2A, RRM2
20	Bhatt et al. [29]	Illum.	6	DMC1, MSH3, IL10, ALPP, HSD17B7, ZNF223,	44	Jiang et al. [55]	Affy.	9	ANLN, BUB1B, BIRC5, CDC20, CDK1, CDCA5, NCAPG, TOP2A, NEK2
21	Jiang et al. [31]	Affy.	13	TLR1, TLR4, TLR8, TLR7, RIPK2, FOS, YWHAZ, FOSL2, FASLG, HIF1A, CDK1A, CCL4, DDIT3	45	Li et al. [56]	Affy.	16	BIRC5, CCNB2, BUB1, CDC20, CDK1, CDC25C, CXCL12, CEP55, KIF20AK, FOS, NUSAP1, RACGAP, KIF2C, SPC24, PRC1, TOP2A
22	Zhang et al. [30]	Affy.\Illum.	20	CDK1, CDC20, CCNB2, AURKA, CCNA2, CCNB1, MAD2L1, BUB1B, TOP2A, BUB1, IGF1, ESR1, FTCD, C8A, CYP3A4, CYP2E1, SPP2, F9, TAT, CYP2C9	46	Dai et al. [59]	Affy.	20	ANLN, NDC80, DLGAP5, NUSAP1, PBK, RACGAP1, NUF2, BUB1B, ZWINT, CCNB1, DTL, TOP2A, KIF20A, RRM2, CDKN3, PRC1, HMMR, NPY1R, CCL20, CXL12
23	Wu et al. [33]	Affy.	12	TTK, CCNB1, TOP2A, RRM2, PRC1, NCAPG, CDK1, UBE2C, CDKN3, ZWINT, RACGAP1, AURKA	47	Zhu et al. [58]	Affy.	10	UBE2C, TK, CDK1, NCAPG, RAD51AP1, TOP2A, ASPM, DLGAP5, PBK, NUSAP1
24	Mou et al. [24]	Affy.\Illum.	18	TK1, TOP2A, FOS, CDC20, CCNB2, ESR1, CXCL12, VWF, HMMR, FOXO1, ACSM3, ZIC2, RFC4, TXNRD1, COL4A1, CYP3A4, GNAO1, RAP2A	48	Xing et al. [57]	Affy.\Illum.	15	TOP2A, CCNB2, PCNA, AURKA, BUB1, CDKN3, RFC4, CEP55, DLGAP5, PRC1, MCM2, CDC20, TPX2, RACGAP1, MCM4

NHG: Number of Hub Genes

### Identification of KGs

**Fig 10:**
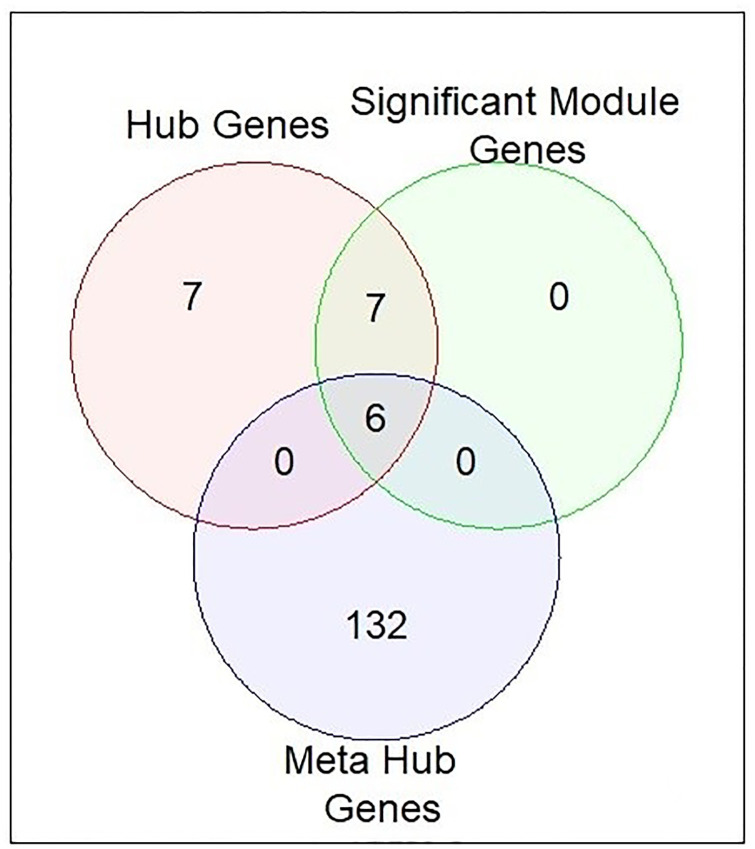
Identification of KGs from hub genes, significant module genes, and meta hub genes.

We determined 20 hub genes from the PPI network using MCC, 13 significant module genes from significant hub modules, and 138 meta hub genes from existing meta hub genes. We identified six common genes among selected hub genes, significant module genes, and meta hub genes as illustrated in Fig 10. The six common genes were CDC20, TOP2A, CENPF, DLGAP5, UBE2C, and RACGAP1, which were assumed to be KGs that can be used to classify patients as having HCC or healthy controls.

### Validation of KGs

#### Discriminative capability analysis using AUC

Four independent datasets (GSE14520, GSE87630, GSE47197, and TCGA-LIHC) were used to validate six KGs. Since these six KGs were identified from datasets with three independent platforms: Affymetrix, Illumina, and Agilent, respectively. We also took these datasets from Affymetrix, Illumina, and Agilent platforms to show the discriminative power of the selected KGs. Moreover, we also checked the discriminative power of these KGs using the TCGA-LIHC dataset. The discriminative power of these six KGs was evaluated using AUC.

**Fig 11:**
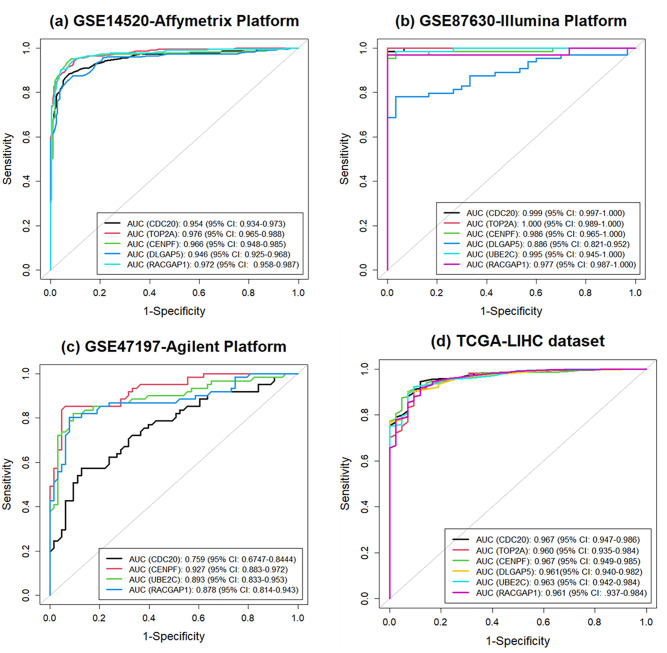
Validation of KGs using AUC: (a) GSE14520; (b) GSE87630; (c) GSE47197, and (d) TCGA-LIHC datasets.

The ROC curves of six KGs for three independent datasets are shown in Fig 11. Five KGs out of six KGs were found in the GSE14520 dataset with the Affymetrix platform (see in Fig 11a), six KGs were found in the GSE87630 dataset with Illumina platform (see in Fig 11b), and four KGs were found in the GSE47197 dataset with the Agilent platform (see in Fig 11c). As shown in Fig 11a, the AUC value of each KG was more than 0.900, which was assumed to be a high discriminative power. So, the correspondence AUC values were 0.954 for CDC20, 0.976 for TOP2A, 0.966 for CENPF, 0.946 for DLGAP5, and 0.972 for RACGAP1. Similarly, as shown in Fig 11b, the AUC values of six KGs were: 0.999 for CDC20, 1.000 for TOP2A, 0.986 for CENPF, 0.886 for DLGAP5, 0.995 for UBE2C, and 0.977 for RACGAP1. Similarly, as shown in Fig 11c, the AUC values of four KGs were: 0.759 for CDC20, 0.927 for CENPF, 0.893 for UBE2C, and 0.878 for RACGAP1 Moreover, the ROC curve of these six KGs for the TCGA-LIHC dataset is shown in Fig 11d. As shown in Fig 11d, we observed that the AUC values of CDC20, TOP2A, CENPF, DLGAP5, UBE2C, and RACGAP1 based KGs were as follows: 0.967, 0.960, AUC: 0.967, 0.961, 0.963, and 0.961, respectively.

#### Survival analysis

**Fig 12:**
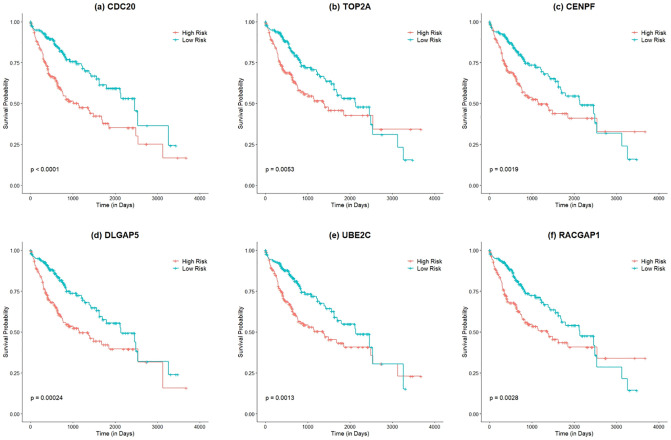
Survival analysis of six KGs using KM plotter: (a) CDC20; (b) TOP2A; (c) CENPF; (d) DLGAP5; (e) UBE2C; and (f) RACGAP1.

The survival analysis of six KGs was performed using univariate Cox regression and their correspondence findings are shown in Fig 12. The patients with HCC were classified into low-risk and high-risk groups and marked as different colors. For example. red line indicates the patients in high risk groups, and the green line indicates the patients in low-risk groups. Specifically, the high expression levels of CDC20 (p<0.0001), TOP2A (p = 0.0053), CENPF (p = 0.0019), DLGAP5 (p = 0.00024), UBE2C (p = 0.0013), and RACGAP1 (p = 0.0028) were determined as being strongly associated with survival status (alive/death). Moreover, the hazard ratio (HR) of these six KGs was greater than one, which indicates there was a strong relationship between these six KGs and the HR of the death. Moreover, HCC patients with over-expression levels of CDC20, TOP2A, CENPF, DLGAP5, UBE2C, and RACGAP1 KGs had significantly poor survival periods than the lower expression level of these genes among patients as shown in Fig 12.

### Necessity analysis of platform-independent KGs identification

**Fig 13:**
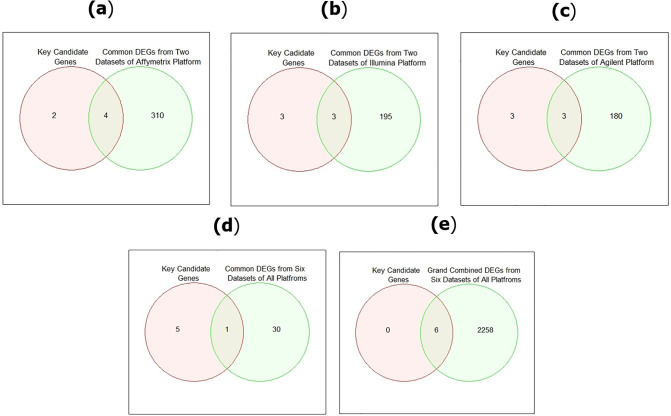
Necessity analysis of KGs on microarray platforms: (a) KGs vs. common DEGs from two datasets of Affymetrix platform; (b) KGs vs. common DEGs from two datasets of Illumina platform; (c) KGs vs. common DEGs from two datasets of Agilent platform, (d) KGs vs. common DEGs from six datasets of all platforms; (e) KGs vs. grand combined DEGs from all platforms.

Six KGs were identified from Affymetrix, Illumina, and Agilent platforms. Now, we performed a necessity analysis to check whether these six identified KGs were present or absent on microarray platforms. Like existing studies, we identified the common DEGs from two datasets of each platform and common DEGs from six datasets of all platforms in order to show whether it is possible to identify these six KGs by following the procedure of existing studies. The result of the necessity analysis of identified six KGs on three microarray platforms is presented in Fig 13. As shown in Fig 13, we observed that only four KGs (TOP2A, CENPF, DLGAP5, and RACGAP1) were identified among common DEGs, obtained from two datasets of Affymetrix platform (see in Fig 13a), three KGs (CDC20, TOP2A, and UBE2C) were found in common DEGs, obtained from two datasets of Illumina platform (see in Fig 13b), three KGs (TOP2A, CENPF, and DLGAP5) were also found in common DEGs, obtained from two datasets of Agilent platform (see in Fig 13c), only one KG (TOP2A) were found in common DEGs, obtained from six datasets of all platforms (see in Fig 13d). On the other hand, six KGs were completely presented in grand combined DEGs, obtained from six datasets of all platforms by following our procedure. From these results, it has been shown that analysis depending on a specific platform (Affymetrix, Illumina, and Agilent) and analysis depending on common DEGs from all platforms can not produce these six KGs.

## Discussion

HCC is one kind of tumor, significantly correlated with poor survival rate and high death rate among most cancer types [89]. The poor survival rate has occurred due to the lack of early detection and the higher incidence rate. In existing studies, researchers have attempted to propose promising biomarkers for detecting HCC. Some of the existing studies conducted their research on genetic datasets that were taken from a particular platform [15, 27], and some researchers used datasets from different platforms [23, 24, 50, 52], but they considered only the common DEGs among those platforms. As a result, both types of studies missed some important genes in their analysis. In order to solve these problems, we have taken datasets from multiple platforms and designed a computational approach with the combination of statistics and ML-based approaches for identifying platform-independent KGs for HCC by considering all important genes.

To identify KGs for HCC, we used six datasets (GSE121248, GSE69715, GSE36376, GSE39791, GSE54236, and GSE115018) from Affymetrix, Illumina, and Agilent platforms. Two datasets were taken from each platform. First, we identified a total of 645 DEGs, 1084 DEGs, 377 DEGs, 279 DEGs, 563 DEGs, and 734 DEGs from GSE121248, GSE69715, GSE36376, GSE39791, GSE54236, and GSE115018 datasets, respectively and their volcano plots and heatmaps of DEGs for individual dataset were presented in Fig 3. After that, we selected 1415 icDEGs from the Affymetrix platform (GSE121248 and GSE69715) (see in Fig 4a), 458 icDEGs from the Illumina platform (GSE36376 and GSE39791) (see in Fig 4b), and 1113 icDEGs from the Agilent platform (GSE54236 and GSE115018) (see in Fig 4c). At the same time, a total of 135 common DEGs were identified from the Affymetrix, Illumina, and Agilent platforms (See in Fig 5). If we analyzed these 135 common DEGs for the next experiments, some important DEGs may be missed from the analysis. To solve this problem, we considered all 2264 (856+92+203+135+332+28+618) gcDEGs, obtained from all platforms. Moreover, we adopted SVM on gcDEGs and then calculated the accuracy of each gene from all 22264 gcDEGs, obtained from all platforms. If the DEGs were presented in multiple datasets, then compute the average classification accuracy. At the same time, DEGs were sorted based on classification accuracy, and we selected 518 DEGs out of 2264 DEGs because their classification accuracy or average classification accuracy was greater than 95.0% as shown in Fig 6. Enrichment analysis was employed on 518 DEDGs to understand their molecular pathways (see Fig 7). It was noticed that BP-based GO terms were significantly correlated of developing HCC patients, which were also enriched DEDGs, coincided with previous studies.

For example, detoxification of copper ion [36, 46], the stress response to copper ion [36], cellular response to copper ion [36, 90], detoxification of inorganic compound [36], the stress response to metal ion [36], cellular response to zinc ion [16], response to zinc ion [16, 54, 57], response to cadmium ion [16, 50, 54, 57], regulation of cell-cell adhesion [6]. The CC-based GO terms were also statistically enriched with DEDGS and coincided with existing studies, collagen-containing extracellular matrix [40]. For MFs based GO terms, DEDGs were also enriched with top GO terms such as chemokine activity [36, 59].

The KEGG pathways were closely associated with DEDG. Our findings also coincided with previous studies, such as Mineral absorption [42, 46, 50, 51, 54, 91], Arginine biosynthesis [40], Viral protein interaction with cytokine and cytokine receptor [59], Cytokine-cytokine receptor interaction [25, 31, 45], Biosynthesis of amino acids, Proximal tubule bicarbonate reclamation, Alanine, aspartate and glutamate metabolism [40, 55], Complement and coagulation cascades [25, 35, 40], PI3K-Akt signaling pathway [25].

A PPI network was constructed using STRING and Cytoscape and determined the 20 hub genes using MCC as shown in Fig 8. Simultaneously, significant modules and their correspondence PPI netwroks as shown in Fig 9. At the same time, 13 combined genes were determined from module 1 and module 2, treated as significant hub module genes. Moreover, meta data were formed from 48 previous studies [6, 9–59] and identified 138 meta-hub genes by combining all hub genes. Finally, this study proposed six KGs (CDC20, TOP2A, CENPF, DLGAP5, UBE2C, and RACGAP1) by intersecting identified hub genes, significant hub module genes and meta hub genes. After that, three independent test datasets from three independent platforms were used to validate the identified six KGs using AUC. Six KGs provided high discriminative power for differentiating HCC from healthy.

CDC20 plays an essential role in the cell division of humans [92]. CDC20 overexpression was strongly associated with various cancer diseases, like colorectal [93] and breast [94]. Existing research found that the overexpression of CDC20 was highly correlated with the development [95] and prognosis of HCC [96]. This current study also illustrated that CDC20 was a KG that played an important role of developing HCC, as coincided by existing studies [10, 13, 14, 17, 18, 20, 24, 30, 32, 37, 40, 41, 44, 48, 50, 55–57].

TOP2A is a cell cycle-related gene that encodes a DNA topoisomerase, which regulates and modifies the topologic states of DNA during transcription. The overexpression of TOP2A was considered as a key biomarker of various cancers, including ovarian [97] and lung [98]. TOP2A overexpression was significantly related to the progression and poor prognosis of HCC [99]. Our study also considered TOP2A as a key biomarker for the development and progression of HCC, as supported by previous studies [12, 14, 15, 18, 20–25, 27, 32–36, 39, 41–43, 45, 46, 48, 50, 51, 54–59].

CENPF is a protein that played a significant role in the segregation of chromosomes during the cell cycle. CENPF was significantly linked with numerous cancers, including HCC [100]. Huang et al. [100] proposed the CENPF gene as a novel significant biomarker for HCC [100]. This current study illustrated that CENPF might play a significant role in developing HCC, supported by previous studies [101, 102]

DLGAP5 also plays a vital role in the development and progression of HCC [109, 103]. Liao et al. [104] illustrated that higher expression levels of DLGAP5 were found in HCC patients compared to healthy subjects [104]. Moreover, the higher expression of DLGAP5 was significantly associated with venous permeation and cellular invasion, as coincided by previous studies [9].

Similarly, this study also considered UBE2C as one of the key biomarkers for the development and prediction of HCC as supported by previous studies [10, 18, 33, 36, 41, 44, 58]. A study showed that UBE2C was one of the key promising biomarkers of HCC [105]. RACGAP1 is a key regulator in various cancers, like colorectal cancer [106], ovarian cancer [107], and breast cancer [108]. A study showed that RACGAP1 was one of the diagnostic prognostic biomarkers for early developing HCC [109]. Our study also showed that RACGAP1 might play a critical role in the development of HCC. This fining was also coincided with existing studies [35, 38, 39, 43, 57, 59].

To show the discriminative power of these proposed six KGs, We used four independent test datasets. The validation experimental results illustrated that CDC20, TOP2A, CENPF, DLGAP5, UBE2C, and RACGAP1 genes had more discriminative capability to be KGs. Moreover, we also performed the prognosis analysis of these six KGs. Interestingly, the six KGs contained CDC20, TOP2A, CENPF, DLGAP5, UBE2C, and RACGAP1 were significantly correlated to the poor prognosis of HCC. This research will be necessary to conduct more research on the roles played by these six discovered genes of developing HCC. In the future, we will implement the same pipeline or proposed system to analysis of other cancer types. Despite more research ongoing to develop novel biomarkers, our future work will implement the clinical utilization of determined key biomarkers in order to fulfill the real-world demand.

## Conclusion and future work direction

This current work identified CDC20, TOP2A, CENPF, DLGAP5, UBE2C, and RACGAP1 as KGs for HCC using statistical and ML-based approaches. These KGs were more closely linked to HCC and have been identified as biomarkers and targeted therapy in the diagnosis of cancer. This current research will be helpful for the readers as well as physicians to identify the associated molecular pathway of HCC. It is necessary to conduct more experimental studies to validate these KGs for HCC.

We have also plan to use alternative models, such as linear SVM, decision trees, and other machine learning algorithms in our future studies to assess the discriminative ability of the selected key genes. We also will try to validate the verify the reliability of these key genes in our future studies. Moreover, incorporating RNA-Seq data into our study will significantly broaden the scope of the research, offering deeper insights into gene expression regulation. We have plan a subsequent study focused exclusively on RNA-Seq datasets for HCC. This future analysis will allow for a direct comparison of RNA-Seq with the platforms used in this study.
